# Effects of nano-zinc oxide supplementation on milk yield, rumen fermentation, nutrient digestibility, and blood indices of high-yielding dairy cows

**DOI:** 10.3389/fvets.2025.1720270

**Published:** 2025-11-19

**Authors:** Abed Zarghami, Mahdi Ganjkhanlou, Abolfazl Zali, Ashkan Fekri, Valiollah Palangi

**Affiliations:** 1 Department of Animal Science, College of Agriculture and Natural Resources, University of Tehran, Karaj, Iran; 2 Department of Animal Sciences, Faculty of Agriculture, Near East University, Nicosia, Cyprus

**Keywords:** nanoparticles of zinc oxide, milk yield, Holstein dairy cows, enzymes, performance

## Abstract

Nanoparticles of zinc oxide (NP- ZnOs) are the most extensively utilized nanoparticles due to the higher surface area, improved bioactivities, and, most importantly, unique chemical stability and simplicity of production. The study aimed to evaluate the effects of NP-ZnOs on the rumen parameters, total-tract nutrient digestibility, and milk performance of Holstein dairy cows. In a completely randomized design, twenty-four dairy cows (650 ± 20 kg of BW; mean ± SD) were allocated to one of four experimental diets, which were as follows: (1) CON + 30 ppm zinc oxide, (2) CON + 30 ppm ZnO-NPs, (3) CON + 60 ppm ZnO-NPs, and (4) CON + 90 ppm ZnO-NPs. Throughout the experiment period, milk yield and milk composition were recorded automatically at each milking time. Body weight (BW) and body condition score (BCS) were assessed throughout the experiment. The components of milk production were unaffected by the supplemental zinc (all *p* > 0.05). Administration of NP-Zn at 90 ppm caused a numerical decrease in somatic cell count (SCC) as compared to the other experimental treatments (*p* = 0.93). The zinc content of milk increased significantly with NP-ZnO supplementation regardless of the dose (*p* < 0.01). Dairy cows receiving diets supplemented with 30 ppm NP-Zn had higher dry matter intake (DMI), crude fat, and NDF digestibility in comparison the other groups. Compared to the other treatments, Group feeding with NP-ZnO at 90 ppm showed the highest concentrations of glucose (*p* = 0.94) and TG (*p* = 0.43), and group receiving 30 ppm resulted in higher cholesterol (*p* = 0.49). The indicator of inflammation, albumin, showed a similar trend (*p* = 0.41). Total volatile fatty acids (TVFAs) concentration increased with adding NP-Zn at 60 ppm dose (*p* = 0.48), although the ruminal content of NH_3_-N showed a lower value compared to the other doses (*p* = 0.329). In conclusion, these results suggested that supplementing diets of high-yielding dairy cows with NP-ZnOs at 90 mg/kg dose could be a profitable substitute for high dietary ZnO inclusion in diets to improve the productivity of dairy cows.

## Introduction

Trace minerals (TM), such as Zn, Mn, Cu, and Co are required for structural proteins, enzymes, coenzymes, and cellular proteins, and participate in many enzymatic processes (1); this leads to changes in the ruminal environment, affecting the production of volatile fatty acid (VFA), fiber digestibility, and feed digestion (2). Because of quantum mechanics and their high surface area-to-quantity ratio, nanoparticles with sizes between 1 and 100 nm promise great potential for use in a range of sectors, including animal feed (3). In the same way, the trace minerals could modify microbial populations and metabolic pathways in the rumen (2). Studies show a lower cellulose digestion decreases due to TM deficiencies at high levels of starch (4). This can occur because fast-growing bacteria (present in high-grain diets) have a higher TM requirement to degrade starch, and they compete with slow-growing bacteria that are cellulose digesters (4). Additionally, supplementation of diets with TM can also modify the molar proportions of VFA (5). In diets deficient in Se, the ruminal molar proportion of isovaleric acid increased with Se, which is explained by increased activity of seleno-enzymes (1).

A necessary mineral, zinc (Zn), participates in a variety of biological processes, such as gene expression, cell signaling, enzyme function, and integrity of cell membranes (6–8). Almost every metabolic pathway depends on at least one zinc-requiring enzyme, since it functions in the structural and functional integrity of approximately 2000 transcription factors and 300 enzymes (9). In spite of the fact that zinc is typically fed as inorganic salts, zinc oxide (ZnO) and zinc SO_4_, increasing the bioavailability of zinc may increase ruminant productivity (10, 11). NP-ZnOs are feed additives that are beneficial because of their smaller particle size, larger surface area, and higher chemical reactivity than typical sources, based on al (12), NP-ZnO has been applied to a variety of sectors, including animal nutrition (13, 14).

Due to their sufficient bioavailability, nanoparticles of trace minerals can be used to decrease mineral excretion and environmental contamination (15). Despite some research showing that NP-ZnO has hazardous effects on animals (16), other studies support the contradictory outcomes (10, 17). For example, feeding dairy cows with organic trace minerals instead of inorganic sources improved fertility, health, and production performance (18–20). In one study, Formigoni, Fustini (21) found that feeding dairy cows with 500 g/kg of Cu, Mn, and Zn organic trace minerals (as OTM) concentration of milk fat increased by 4.4%, meanwhile the experimental supplements did not have any impact on milk yield, protein concentration, and somatic cell count (SCC). The contradictory findings may result from the purity of the trace mineral supplements, stressors, agents, mineral content in body cells, and the physiological stage of animals (11).

Although zinc oxide (ZnO) is widely used as a dietary supplement in dairy nutrition, there is a lack of adequate research regarding the effects of nano-sized ZnO (NP-ZnO) on the performance of high-yielding Holstein dairy cows. Given that nanoparticle forms may offer higher bioavailability and enhanced cellular uptake compared to conventional ZnO, it is rational to hypothesize that NP-ZnO could improve the metabolic efficiency and productive performance of dairy cows. Therefore, this study was designed to evaluate the effects of NP-ZnO supplementation on milk yield and composition, blood metabolites, nutrient digestibility, and rumen fermentation characteristics in Holstein dairy cows.

## Methods and materials

### Characteristics of NP-ZnOs

An apparatus for grinding (Just Nanotech Co., JBM-B035, Tainan, Taiwan) was used to produce NP-ZnO. 240 milliliters of 95% ethanol were mixed with a mixture of dry materials consisting of 2.5 grams of dispersed reagent-silica and 10 grams of zinc oxide to create a slurry. After 1.5 h of pre-mixing, the slurry was put in a grind chamber containing 500 g of 0.2 mm zirconium particles. Next, the mixture was milled at 960 × g for 1.5 h. Following grinding, big particles were eliminated from the slurry by passing it through a 200-mesh sieve. Following that, the slurry was oven-dried for a whole night at 50 °C. After that, a 200 mesh sieve was again used to filter the NP-ZnO powder. The zinc concentration of NP-ZnO was determined using the atomic absorption spectrometer (Perkin Elmer, Atomic Analyst 100, Waltham, MA, USA).

### Animal and experimental design

This study was carried out at the airy research Center of the University of Tehran, Iran. The University of Tehran’s Animal Science Committee gave its approval for the animal care and use (No. 9330381002–28.09.94). A total of twenty-four multiparous lactating Holstein dairy cows (650 ± 20 kg of BW; 70 ± 4 DIM; parity 2 to 3) in completely randomized design were randomized to receive one of the following four treatments: (1) CON + 30 ppm zinc oxide, (2) CON + 30 ppm ZnO-NPs, (3) CON + 60 ppm ZnO-NPs, and (4) CON + 90 ppm ZnO-NPs. The diets containing supplementary minerals had higher zinc concentrations compared to the control diet. Cows were placed in separate box stalls (4.40 × 4.40 m^2^) and had free access to water throughout the study. Milking of the cows was taken three times a day at 0600, 1300, and 1900, and recorded at each milking time. To determine the change in BW, the animals were weighed at the start, middle, and end of the trial, soon after the afternoon milking. BCS were assessed using the protocols described by Arruda, Godden (22), during the same time tables used for BW measures. Dietary ingredients were combined and supplied as TMR on a daily basis at 0700 and 1700 h. Orts were weighed and collected daily before the afternoon feeding. Individual feed intake was recorded throughout the experiment, and the amount of feed offered was modified every day to obtain 5 to 10% orts. Table 1 describes the ingredients and nutritional composition of the base diets (NRC, 2001).

**Table 1:** Ingredient and chemical composition of experimental diets (g/kg DM unless noted otherwise).

	Experimental diets^a^			
Ingredient				
Corn silage	25.4	25.4	25.4	25.4
Alfalfa hay, chopped	15.9	15.9	15.9	15.9
Corn grain, ground	17.2	17.2	17.2	17.2
Barley grain, ground	7.93	7.93	7.93	7.93
Soybean meal	6.95	6.95	6.95	6.95
Wheat bran	13.5	13.5	13.5	13.5
Canola meal	10.4	10.4	10.4	10.4
Calcium salts of fatty acids^b^	1.16	1.16	1.16	1.16
Dicalcium phosphate	0.12	0.12	0.12	0.12
Sodium bicarbonate	0.58	0.58	0.58	0.58
Vit and min mix^c^	0.42	-	-	-
Vit and min mix	-	0.30	-	-
Vit and min mix	-	-	0.60	-
Vit and min mix	-	-	-	0.90
Magnesium oxide	0.12	0.12	0.12	0.12
Bentonite	0.19	0.19	0.19	0.19
Salt	0.24	0.24	0.24	0.24
Chemical composition				
DM (%)	52.2	52.2	52.2	52.2
CP (%)	17.9	17.9	17.9	17.9
NDF^d^ (%)	32.1	32.1	32.1	32.1
NFC^e^ (%)	36.3	36.3	36.3	36.3
NE_L_^f^ (Mcal/kg)	1.71	1.71	1.71	1.71
Starch (%)	26.7	26.7	26.7	26.7
Ash (%)	7.13	7.13	7.13	7.13

^a^Experimental diets were: (1) CON + 30 ppm zinc oxide, (2) CON + 30 ppm ZnO-NPs, (3) CON + 60 ppm ZnO-NPs, and (4) CON + 90 ppm ZnO-NPs.

^b^Ca-salts of fatty acids, Persiafat, Kimiya Danesh Alvand Co. Iran., contained 84% fat and 9% Ca (1% C14:0; 28% C16:0; 3% C16:1; 5% C18:0; 26% C18:1; 30% C18:2; 3% C18:3; 3% other fatty acids).

^c^Each kilogram of mineral and vitamin supplement mix contains: calcium: 170 grams, phosphorus 60 grams, magnesium: 100 grams, manganese: 13000 mg, copper: 5000 mg, iron: 4000 mg Cobalt: 80 mg, Selenium: 110 mg, Iodine: 200 mg, Vitamin A: 1,250,000 IU, Vitamin D3: 300,000 IU, Vitamin E: 6000 IU. ZnO-NPs (zinc oxide nanoparticles with a purity of more than 99%, containing 79% zinc).

^d^Neutral detergent fiber exclusive of residual ash.

^e^Was calculated as 100 – (NDF + CP + Ether extract + Ash).

^f^Calculated according to NRC (2001).

### Milk sampling and analysis

Milk samples were placed in tubes preserved with 2-bromo2-nitropropane-1,3 diol (Broad Spectrum Microtabs II; Advanced Instruments Inc.) and kept at −20 °C until they were shipped overnight to Vahdat Cooperative Inc. (Isfahan, Iran) laboratory. To determine fat, true protein, lactose, SCC, and milk urea nitrogen (MUN), Fourier transform infrared spectroscopy with a MilkoScan FT + (Foss Inc.) was utilized. Milk yield and components were calculated for each cow individually; the following formulas were utilized to calculate 3.5% FCM and ECM:


3.5%FCM=[0.4324×milk yield(kg/d)]+[16.216×fatyield(kg/d)];

$$\begin{array}{c} \mathrm{ECM}=0.327\times \text{milk yield}\;(\mathrm{kg}/d)+12.95\times \mathrm{fat}\;\text{yield}\;(\mathrm{kg}/d)\\ +7.2\times \text{protein yield}\;(\mathrm{kg}/d). \end{array}$$

### Blood sampling and analysis

Blood samples (10 mL) from each cow were taken twice a week via the jugular vein and placed into evacuated tubes (MediPlus, Sunphoria Co., Ltd., China) containing EDTA, approximately three hours after morning feeding. Subsequently, blood samples were centrifuged at 2,000 × g for 10 min and frozen at −20 °C until the analysis of biochemical components. To measure serum zinc, 1 mL serum sample was mixed with 4 mL of nitric acid (65%; Sigma, Steinhein, Germany) and perchloride acid (70%; Sigma, Merck, Germany) in a ratio of 3:2. Before being diluted with 1,000 mL of de-ionized water and filtered, the solution was heated to 300 °C until it turned clearer. Following that, the solution was analyzed using an atomic absorption spectrometer (Perkin Elmer, Atomic Analyst 100). Commercially available kits were utilized to measure the levels of serum BUN, glucose, protein, cholesterol, and albumin according to the manufacturer’s instructions (Pars Azmon, Iran). The Biorex kit method (BiorexFars, Iran) included utilizing an atomic absorption spectrophotometer to measure the activity of superoxide dismutase (SOD) after washing red blood cells three times with a 0.9% saline solution (Varian SpectrAA220, Australia). The level of this reaction’s inhibition was then used to gauge SOD activity (23). Each sample’s levels of antioxidant enzymes were measured and expressed as units per gram of hemoglobin (Hb).

### Feed sampling and analysis

To modify TMR and calculate DMI, representative samples (about 300 g) of silage, TMR, and orts were taken twice weekly and dried in a forced-air oven at 55 °C for 48 h. To assay the index of digestibility, spot fecal samples were taken weekly during the experiment (4 samples/cow); were crushed to pass through a 1-mm Wiley mill screen (Arthur H. Thomas), the samples were evaluated for DM, ash, OM, and total N employing a Nitrogen Analyzer (Leco Instruments Inc.); heat-stable *α*-amylase and Na_2_SO_3_ were used to analyze NDF (24). Total-tract apparent digestibility (%) was calculated for all nutrients analyzed as follows:


$$\begin{array}{l} 100-\{\begin{array}{l} \left[\text{nutrient}\%\text{feces}\times \text{fecal output}\;(\mathrm{kg}/d)\right]\\ /[\text{nutrient intake}\;(\mathrm{kg}/d)] \end{array}\},\\ \text{where nutrient intake}\;(\mathrm{kg}/d)=[\begin{array}{l} \text{nutrient}\%\mathrm{TMR}\\ \times \mathrm{TMR}\;\text{offered}\;(\mathrm{kg}/d) \end{array}]\\ -[\text{nutrient}\%\text{orts}\times \text{orts}\;(\mathrm{kg}/d)]. \end{array}$$

### Rumen sampling and analysis

On the last day of the study, approximately four hours after the morning feeding, ruminal fluid (9.8 mL) was collected using a stomach tube (25). After filtering the rumen liquor using three layers of cheesecloth, the ruminal fluid’s pH was quickly determined using a portable digital pH meter, calibrated at pH 4 and 7 (HI 8318; Hanna Instruments, Cluj-Napoca, Romania). The ruminal fluid (9.8 mL) was put into 10-mL centrifuge tubes, acidified with 0.2 mL of a 50% H2SO4 solution (vol/vol), and centrifuged at 7,000 × g for 15 min at 4 °C. Then, the supernatant was kept at −20 °C for further investigation. A Merck Hitachi Elite LaChrome HPLC system (L2400, Hitachi) and a Bio-Rad Aminex HPX-87H column (Bio-Rad Laboratories) were used to evaluate volatile fatty acids. A UV detector (wavelength 210 nm; Hitachi L2400) and a flow rate of 0.7 mL/min at 46 °C were utilized in an isocratic elution system with the column, which included 0.015 M sulfuric acid (H_2_SO_4_) in the mobile phase of HPLC. Ammonia-N was tested with a Technicon autoanalyzer, which used a colorimetric approach to quantify N.

### Statistical analysis

All weekly data were analyzed using SAS version 9.4’s MIXED approach (SAS Institute Inc., Cary, NC) in line with the repeated measures model specified below:


Yijk=μ+Ti+Ak+Ti×Aj+Cj(Ti)+eijk.

where Y_ijk_ = dependent continuous variable, *μ* = overall mean, T_i_ = fixed effect of treatment, A_k_ = fixed effect of time, T_i_ × A_k_ = interaction between treatment and time, C_j_(T_i_) = random effect of cow, and e_ijk_ = residual error. For all variables, the covariates for dry matter intake (DMI) and prior 305-day milk yield were kept in the model when they were significant (*p* < 0.05). Normal probability and box plots were used to confirm that the residuals were normally distributed. The model without the time impact was used to analyze cumulative milk yield and DMI using SAS’s MIXED technique. Significance was set at *p* < 0.05, and trends were considered significant at *p* ≤ 0.10.

## Results

### Milk yield, milk composition, and DMI

**Figure 1:**
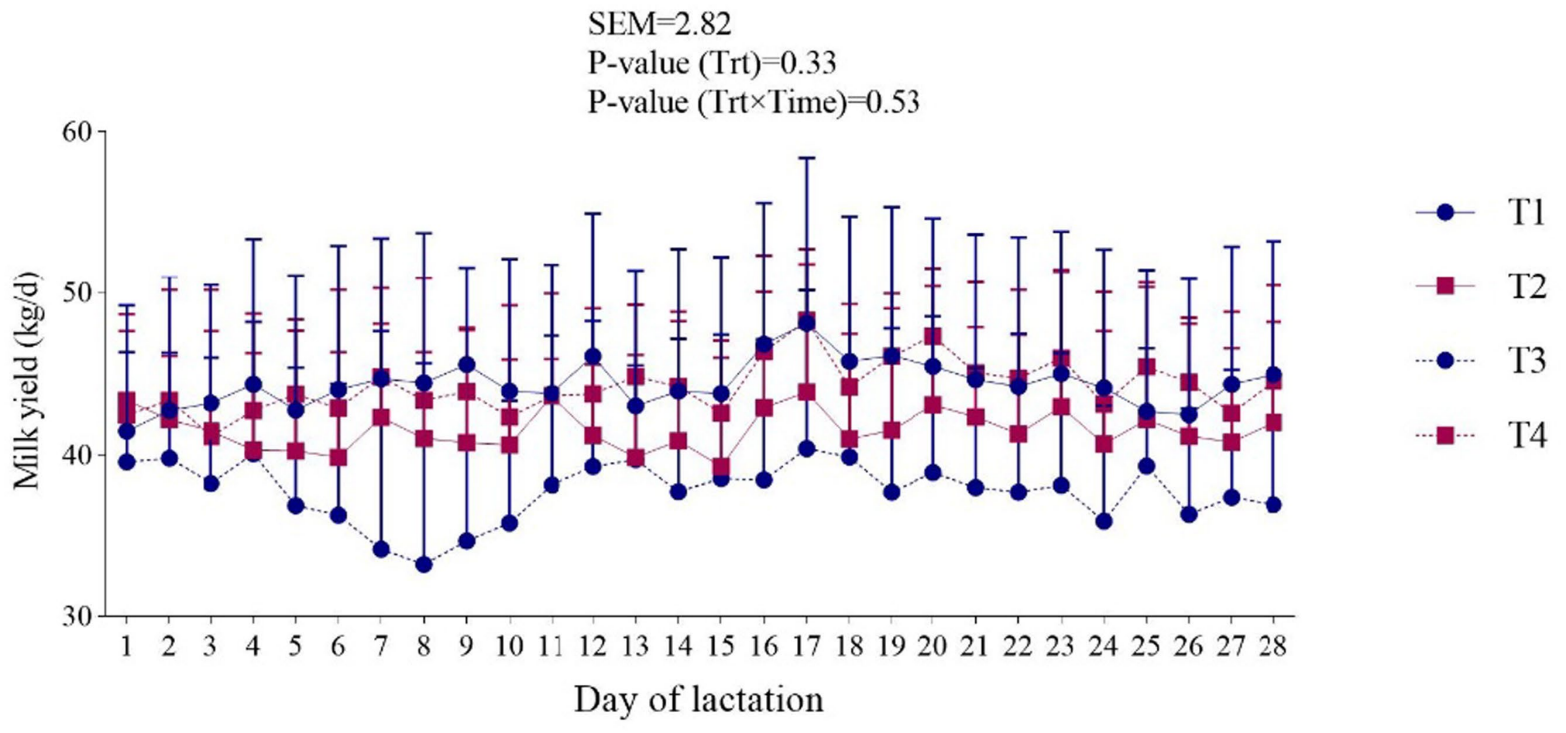
Effect of dietary-supplemented different zinc sources on milk yield of dairy cows. Experimental diets were: (1) CON + 30 ppm zinc oxide, (2) CON + 30 ppm ZnO-NPs, (3) CON + 60 ppm ZnO-NPs, and (4) CON + 90 ppm ZnO-NPs. Values are means, with SEM represented by vertical bars.

Milk compositions are shown in Table 2 and Figure 1. Dry matter intake (DMI) was significantly influenced by the treatments; diets containing 30 ppm NP-ZnO showed a higher value (23.12 kg/d; *p* < 0.01). We observed the highest concentration of Zn in milk with NP-ZnO at 90 ppm dose compared to the other doses (2.71 μg/mL; *p* < 0.01). Regarding the percentage of milk protein and fat, the group receiving 60 ppm NP-ZnO level showed a higher concentration than CON (3.16 and 3.06%, respectively).

**Table 2:** Effect of dietary-supplemented different zinc sources on DMI, milk yield, milk composition, and BW and BCS changes.

	Treatment^a^					*P*-value^b^		
Item^c^	1	2	3	4	SEM	Treatment	Time	Treatment × Time
Intake, kg/d								
DM	22.1	23.1	23.02	22.5	1.13	0.36	<0.01	<0.01
Yield, kg/d								
Milk	44.4	41.5	37.8	44.2	2.82	0.33	<0.01	0.53
3.5% FCM	41.5	39.6	36.4	43.5	2.96	0.39	<0.01	0.55
3.5% ECM	41.6	39.7	36.6	43.5	2.78	0.36	<0.01	0.55
Fat	1.37	1.33	1.24	1.50	0.12	0.45	<0.01	0.56
Protein	1.29	1.23	1.15	1.33	0.08	0.38	<0.01	0.56
Lactose	2.04	1.91	1.72	2.02	0.13	0.33	<0.01	0.53
Milk composition, %								
Fat	3.31	3.17	3.62	3.01	0.23	0.41	0.05	0.92
Protein	2.97	2.98	3.06	3.00	0.09	0.90	0.48	0.93
Lactose	4.58	4.61	4.55	4.57	0.04	0.82	<0.01	0.74
MUN, mg/dL	10.8	10.7	10.6	10.7	0.16	0.71	<0.01	0.16
SCC, 10^3^ cells/mL	2	5	5	3	1	0.69	0	0.9
Zn, μg/mL	0	1	2	2	0	<0.01	-	-
BW change, kg	−18.6	−18.1	−17.8	−14.1	2.33	0.52	-	-
BCS change, kg	2.61	2.71	2.68	2.58	0.07	0.31	-	-

^a^Experimental diets were: (1) CON + 30 ppm zinc oxide, (2) CON + 30 ppm ZnO-NPs, (3) CON + 60 ppm ZnO-NPs, and (4) CON + 90 ppm ZnO-NPs.

^b^*p*-values refer to the ANOVA results for the main effect of treatment, the main effect of time, and the interaction between treatment and time.

^c^3.5%FCM = [0.4324 × milk yield (kg/d)] + [16.216 × fat yield (kg/d)].

^c^3.5% ECM = 0.327 × milk yield (kg/d) + 12.95 × fat yield (kg/d) + 7.2 × protein yield (kg/d).

^c^DM, dry matter; FCM, fat corrected milk; ECM, energy corrected milk; MUN, milk urea nitrogen; SCC, somatic cell count; BW, body weight; BCS, Body condition score.

### Total-tract nutrient digestibility

Dietary supplements did not affect the nutrient digestibility of the dairy cows (Table 3) despite numerical differences across treatments. The 30 ppm NP-ZnO dose had higher dry matter (*p* = 0.14), crude fat (*p* = 0.55), and NDF digestibility (*p* = 0.11); the 60 ppm NP-Zn group had the highest crude protein digestibility (*p* = 0.40).

**Table 3:** Effect of dietary-supplemented different zinc sources on nutrient digestibility of Hosltein dairy cows.

	Treatment^a^					
Item^c^	1	2	3	4	SEM	*P*-value^b^
DM (%)	72.3	73.6	72.5	71.03	0.88	0.145
NDF (%)	58.8	55.4	52.9	53.6	0.62	0.112
CP (%)	77.1	79.2	80.6	81.4	0.45	0.401
EE (%)	70.2	74.4	71.8	69.4	0.92	0.553

^a^Experimental diets were: (1) CON + 30 ppm zinc oxide, (2) CON + 30 ppm ZnO-NPs, (3) CON + 60 ppm ZnO-NPs, and (4) CON + 90 ppm ZnO-NPs.

^b^*p*-values refer to the ANOVA results for the main effect of treatment, the main effect of time, and the interaction between treatment and time.

^c^DM, dry matter; NDF, neutral detergent fiber; CP, crude protein; EE, ether extract.

### Plasma indices

**Figure 2:**
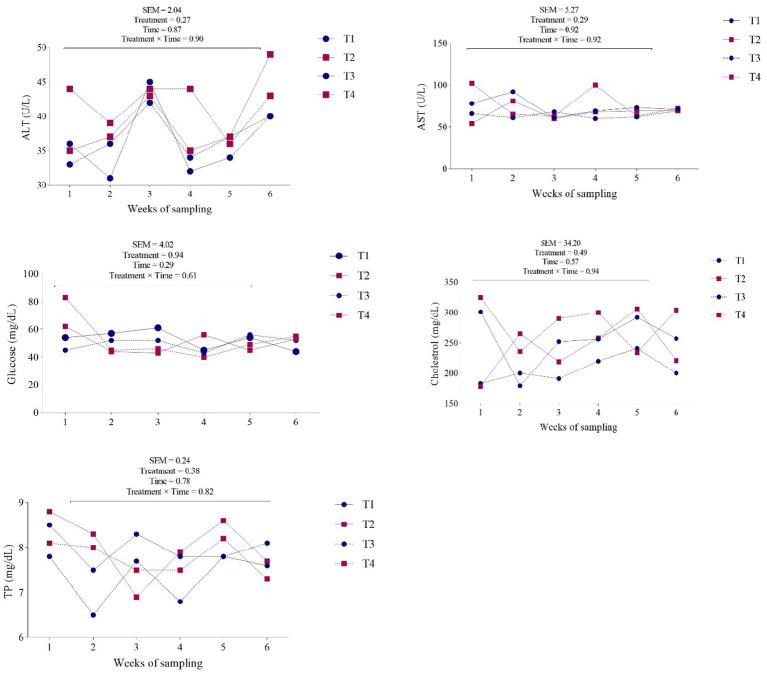
Effects of dietary supplementation with different zinc sources on blood traits of ALT, AST, glucose, cholesterol, and TP in dairy cows. Experimental diets were: (1) CON + 30 ppm zinc oxide, (2) CON + 30 ppm ZnO-NPs, (3) CON + 60 ppm ZnO-NPs, and (4) CON + 90 ppm ZnO-NPs. Values are means, with SEM represented by vertical bars.

Table 4 and Figure 2 present the impact of dietary supplements on plasma biomarkers. Group feeding with NP-ZnO at 90 ppm showed the highest concentrations of glucose (*p* = 0.94) and TG (*p* = 0.43), and the group receiving 30 ppm resulted in higher cholesterol (*p* = 0.49). The indicator of inflammation, albumin, showed a similar trend (*p* = 0.41).

**Table 4:** Effects of dietary-supplemented different zinc sources on blood traits of Hosltein dairy cows.

	Treatment^a^					*P*-value^b^		
Item^c^	1	2	3	4	SEM	Treatment	Time	Treatment × Time
Glucose (mg/dL)	52.5	50.5	50.0	53.0	4.02	0.94	0.29	0.61
Triglyceride (mg/dL)	19.3	18.6	16.1	19.1	1.53	0.43	0.87	0.11
Cholesterol (mg/dL)	256	241	206	189	34.2	0.49	0.57	0.94
Total protein (mg/dL)	7.92	8.03	7.45	7.76	0.24	0.38	0.78	0.82
BUN (mg/dL)	35.3	32.5	35.3	35.7	2.51	0.79	0.35	0.51
Albumin	3.92	3.87	3.13	3.67	2.65	0.41	0.20	0.42
ALT (U/L)	36.3	39.3	37.04	41.6	2.04	0.27	0.87	0.90
AST (U/L)	74.0	66.8	74.3	77.5	5.27	0.29	0.92	0.92
Bilirubin (mg/dL)	0.34	0.35	0.29	0.24	0.05	0.34	0.66	0.67
WBC (10^3^/m^3^)	11.2	11.9	11.2	11.02	0.524	0.13	-	-
RBC (10^3^/m^3^)	8.28	8.45	8.64	9.04	0.617	0.09	-	-
Alkaline Phosphatase (IU/L)	104	106	108	106	5.03	0.184	-	-
SOD (IU g Hb)	2	2	2	2	5	<0.01	-	-
Zn (μg/mL)	1.13	1.26	1.39	1.50	0.09	0.32	-	-

^a^Experimental diets were: (1) CON + 30 ppm zinc oxide, (2) CON + 30 ppm ZnO-NPs, (3) CON + 60 ppm ZnO-NPs, and (4) CON + 90 ppm ZnO-NPs.

^b^*p*-values refer to the ANOVA results for the main effect of treatment, the main effect of time, and the interaction between treatment and time.

^c^AST, aspartate aminotransferase; ALT, alanine aminotransferase; SOD, super oxide dismutase.

### Rumen fermentation parameters

Table 5 shows the characteristics of rumen fermentation in Holstein dairy cows fed with different doses of NP-ZnO supplements. The treatments impacted none of the measures; adding NP-ZnO to the control diet increased total VFA (*p* = 0.48), but a numerical reduction in ruminal content of NH_3_-N (*p* = 0.32). Furthermore, neither the pH (*p* = 0.11) nor the proportions of individual VFAs were impacted by the experimental diets.

**Table 5:** Effect of dietary-supplemented different zinc sources on ruminal pH and fermentation characteristics.

	Treatment^a^					
Item^c^	1	2	3	4	SEM	*P*-value^b^
pH	6.42	6.37	6.22	6.35	0.25	0.116
N-NH_3_ (mg/dL)	17.2	15.4	14.01	16.3	1.46	0.329
TVFA (mmol/L)	114	115	117	115	1.72	0.485
Acetate (%)	68.1	65.5	69.2	66.7	2.98	0.392
Propionate (%)	19.9	20.4	18.6	18.5	0.12	0.465
Butyrate (%)	10.3	8.73	9.12	8.45	0.36	0.097
A/P	3.42	3.21	3.71	3.60	0.09	0.078

^a^Experimental diets were: (1) CON + 30 ppm zinc oxide, (2) CON + 30 ppm ZnO-NPs, (3) CON + 60 ppm ZnO-NPs, and (4) CON + 90 ppm ZnO-NPs.

^b^*p*-values refer to the ANOVA results for the main effect of treatment, the main effect of time, and the interaction between treatment and time.

^c^TVFA, total volatile fatty acids.

## Discussion

### Milk yield and composition, DMI

Supplementing various forms of Zn has resulted in different performances of Holstein dairy cows. During the period, there was an increase in DMI of dairy cows fed with different doses of NP-ZnO, ranging from 30 mg/kg DM to 90 mg/kg DM. In their study, Raje et al. (15) observed an increase in DMI in pre-weaning dairy calves receiving different doses of Zn in the starter (35.5, 34.7, or 33.7 mg/kg of DM) and attributed it to the higher bioavailability of the Zn source. The higher DMI in rats (26) and broilers (27) fed with nano-particles of Zn in their diets. Additionally, Wang et al. (28) showed that high-yielding Holstein dairy cows fed with diets supplemented with coated zinc sulfate (CZS) increased their DMI as compared to the group fed with CON diets. The authors suggested that the higher DMI is probably a consequence of higher digestibility of NDF and ADF, which increases the passage rates of the fiber (29), although in the current trial, we did not observe any alterations in NDF digestibility. This finding is in disagreement with the outcomes of Cope et al. (18) who showed that different levels of ZnO (40 and 60 mg/kg DM) in a 14-wk trial did not influence DMI of dairy cows. However, in a study of Malcolm-Callis, Duff (30) there was a reduction in DMI of cross-bred beef steers (British × Continental) that received an increasing dose of ZnSO_4_. The decrease in DMI was related to the adverse impacts of higher ZnO doses, although the form of Zn had a negligible effect on the DMI of dairy cows (18).

It has been stated that the higher DMI and DM digestibility (DMD), critical parameters influencing animal performance, were the causes of the positive impact of zinc supplementation on milk production, regardless of the type of zinc supplement (31, 32). Adequate administration of Zn in the diets of dairy cows has the potential to improve productivity by reinforcing the intestinal epithelial membrane, stabilizing the microbiota in the gut, optimizing the efficiency of digestion and absorption of nutrients, and increasing the availability of nutrients and energy for higher milk production (33–35). Furthermore, Xie et al. (35) stated that an increase in milk production by incorporating Zn sources into diets at an appropriate dose might have a beneficial impact on milk efficiency of dairy ruminants. Milk production was unaffected by the different doses of NP-ZnO in the current investigation (Table 3), which is in line with the findings of Martino, Ianni (36), who stated that lack of influence of ZnO or NP-ZnO at 44 mg/kg DM was likely caused by the fact that the bioavailability of supplemental Zn may not be important to show a significant effect on milk yield (37). Furthermore, in a study, Ianni, Innosa (38) did explore no increase in DMI and milk yield of Friesian dairy cows fed with 37 mg/kg DM. Comparing dietary zinc in organic and inorganic forms, Cope, Mackenzie (18) found that although DMI did not change among the treatments, milk yield increased with 300 mg of chelated Zn/kg DM in the diet of Holstein-Friesian dairy cows; however, milk components were not influenced by the Zn sources and doses. However, Wang et al. (28) observed an increase in milk yield in groups of dairy cows fed with Zn sources and linked to factors, such as Zn concentration in basal diets, dose, and sources of Zn administered into the diets.

There have been suggestions that the ewes receiving Zn supplements, particularly NP-ZnO, had lower milk SCC, in contrast to the control group. This could be because the animals fed ZnO and NP-ZnO had better total leukocytes and total antioxidant capacity (TOAC), which reduces the udder’s vulnerability to bacterial infections (18, 39). Another possible explanation for NP-ZnO’s improved efficacy in lowering ewe milk SCC is its diminished impact on harmful microorganisms (40). Additionally, Zn exhibits antioxidant properties and stabilizes membranes, which suggests its role in the prevention of free-radical-induced injury during inflammatory processes (41). In another study, Rajendran, Kumar (42) found that in comparison to animals fed ZnO, supplementation of diets with NP-ZnO enhanced the production of milk and reduced milk SCC in cows with subclinical mastitis. Also, Machado, Bicalho (43) showed that injection of a multimineral preparation (including selenium, copper, zinc, and manganese) had a positive impact on udder health, decreasing linearly SCC scores and the incidence of subclinical and clinical mastitis. In contrast, Whitaker, Eayres (44) recorded no effect of dietary zinc supplementation on SCC. Furthermore, Spain (45) found no significant differences in SCC in the Iris trial, whereas in fact the cows receiving zinc proteinate ended up with slightly higher values. According to Chibisa, Christensen (46), normal ruminal fermentation, which prevents fluctuations in milk fat, may be the reason for the lactating ewes’ identical milk fat contents, suggesting that the ewes’ ability to obtain post-ruminal metabolizable protein (MP) was unaffected by the various dietary zinc sources, as there was no influence of either ZnO or NP-ZnO on the milk protein. Raynal-Ljutovac, Lagriffoul (47) found that the zinc level of CON milk was similar to that of raw milk. The Zn content of milk was also found to be substantially raised by a Zn-enriched diet (36), which is in line with the current study.

### Total-tract nutrient digestibility

The nutrient digestibility results were consistent with those of Mandal, Dass (48), who found that in bulls fed a basal diet (32.5 mg Zn/kg DM) supplemented with 35 mg Zn/kg DM, dietary zinc did not affect DM, CP, ADF, or NDF digestibility. Likewise, Garg, Mudgal (49) found no impact of zinc supplementation on lambs’ N retention or balance. Furthermore, piglets (50) and goat kids (51) did not exhibit a change in DMD when fed NP-ZnO instead of ZnO. However, Salama, Caja (52) found that dairy goats fed a diet supplemented with 1 g/day of organic zinc had higher apparent digestibility of DM and CP. Furthermore, Garg, Mudgal (49) discovered that feeding lambs a diet with 34 mg of zinc/kg DM in addition to 20 mg of organic zinc/kg DM enhanced the digestibility of ADF while not affecting DM, CP, or NDF digestibility. The therapeutic benefits of zinc supplementation on DMD in the studies may be attributed to the beneficial effects of zinc on the activities of digestive enzymes (53) and the proliferation and function of rumen bacteria (54). An additional explanation could be related to how zinc improves the gastrointestinal tract’s hydrolase activity (55). It might be connected to the increased proliferation of fiber-degrading rumen bacteria due to NP-ZnO’s great adsorbing capacity and higher surface activity (56). In line with these studies, the other researchers demonstrated that NP-ZnO supplementation improved digestibility both *in vitro* (57) and *in vivo* studies (58), and related it to the stimulation of rumen microbial activity and the subsequent biological action of nano-minerals. It is crucial to consider that preparation and time affect the stability and size of nanoparticles. Therefore, it is preferable to determine the nano minerals’ particle size right before the experiment.

### Plasma indices

The variations in SOD activity in plasma between the treatments were statistically significant. Additionally, NP-ZnO increased several enzyme activities, such as alkaline phosphatase. According to Li, Huang (50), zinc has an essential role in SOD, the main antioxidant enzyme found in cells, which is involved in detoxifying superoxide free radicals and shielding cells from oxidative stress. Bun, Guo (59) reported an increased SOD in the plasma and liver of broilers with increasing the levels of supplementation and the use of organic sources. It is reported that Zn is an essential component of SOD, the primary antioxidant enzyme in cells, which plays a fundamental role in the detoxification of superoxide free radicals and protection of cells against oxidative stress (60). Zn deficiency leads to decreased activity of SOD, resulting in increased tissue sensitivity to oxidation due to the weakness of the antioxidant system (10). One of the proposed mechanisms of action for Zn is its capacity to displace transition metals (Fe and Cu) from binding sites. Zn can compete with iron and copper to bind to the cell membrane and decrease the production of free radicals, thus exerting a direct antioxidant action (41).

Compared to ZnO, feeding NP-ZnO resulted in a decrease in white blood cells, which might be NP-ZnO has a higher cellular bioavailability (61). Similarly, the dietary substitution of NP-ZnO for ZnO demonstrated a positive impact on the piglets’ immune systems (50). According to NRC (2001), ruminants typically have plasma zinc levels between 0.9 and 1.5 mg/L. Current results demonstrated no discernible variation in the plasma zinc content between these groups, and that dietary supplementation of NP-ZnOs raised plasma zinc concentration, demonstrating that NP-ZnO was more accessible than inorganic ZnO.

According to previous reports, nanoparticles can improve drug absorption and have a greater bioavailability (54, 62). Comparable to our findings, Chhabra and Arora (63) discovered that goats given a basal diet (15 mg Zn/kg DM) supplemented with 65 mg Zn/kg DM had greater plasma Zn contents than control goats. In addition, Puchala, Sahlu (64) observed that Angora goats fed a basal diet containing 22 mg Zn/kg DM had greater serum Zn concentrations when supplemented with 40 mg Zn/day. According to Jia (65), when zinc levels are at a medium level, homeostatic mechanisms would not expect supplementary zinc to raise plasma zinc concentrations. This suggests that the ideal supplemental zinc concentration for Cashmere goats was 30 mg/kg DM (total of dietary zinc 52.3 mg/kg DM).

### Rumen fermentation parameters

The ruminal pH values of the cows ranged from 6.22 to 6.42, which falls within the typical range of 5.5 to 6.8 (32). The concentration of NH_3_-N varied from 14.01 to 17.23 mg/dL, exceeding the minimum concentration of 5 mg/dL required for the proper establishment of rumen bacteria (32). Due to the denaturing effect of higher diet zinc on soluble proteins and the deactivating effect of supplemental zinc on ruminal proteolytic enzymes, the dietary addition of NP-ZnOs may have reduced ruminal NH_3_-N concentration by decreasing ruminal proteolysis (66). Another explanation for the lower ruminal NH_3_-N could be the increased intake, digestibility, and energy availability, which also promote the absorption of ammonia in microbial protein (67). The higher DMI and DMD of the Zn-supplemented cows may be a result of higher ruminal TVFA content compared with the control group (32). Nevertheless, the identical molar ratios of the distinct VFA in the cows’ rumen demonstrated that the dietary Zn supplementation did not affect their relative production and absorption. According to the current findings, adding Zn sources to the diet may alter the rumen fermentation process to capture the increased feedstuff energy as VFA. These statements are consistent with the results of Bateman II, Williams (68). Similarly, Kumar (57) demonstrated that adding NP-ZnOs to a diet based on sorghum stover raised the *in vitro* ruminal VFA concentration while not affecting the number of protozoa. Ginting and Simanihuruk (69), on the other hand, found that giving goats higher dietary quantities of zinc, such as ZnO, did not affect their ruminal VFA.

## Conclusion

In conclusion, the current study demonstrates that supplementation with NP-ZnO improved Zn concentration in both milk and serum compared with the control diet. The results also indicate a potential positive role of NP-ZnO in enhancing milk composition, Zn retention, and certain blood antioxidant parameters in dairy cows. Although these improvements were not always statistically significant, the overall numerical trends suggest a beneficial effect on physiological responses. Considering milk performance, a dietary inclusion level of 90 ppm NP-ZnO appears to be a promising concentration. Nevertheless, further studies with larger sample sizes and longer feeding periods are needed to confirm these findings and to clarify the absorption mechanisms and metabolic pathways of NP-ZnO in dairy cows.

## Data Availability

The original contributions presented in the study are included in the article/supplementary material, further inquiries can be directed to the corresponding authors.
